# ATG5 Promotes Death Signaling in Response to the Cyclic Depsipeptides Coibamide A and Apratoxin A

**DOI:** 10.3390/md16030077

**Published:** 2018-03-01

**Authors:** Xuemei Wan, Jeffrey D. Serrill, Ian R. Humphreys, Michelle Tan, Kerry L. McPhail, Ian G. Ganley, Jane E. Ishmael

**Affiliations:** 1 Department of Pharmaceutical Sciences, College of Pharmacy, Oregon State University, Corvallis, OR 97331, USA; wanxu@oregonstate.edu (X.W.); jeffrey.serrill@gmail.com (J.D.S.); humphrei@oregonstate.edu (I.R.H.); mwgstan@gmail.com (M.T.); kerry.mcphail@oregonstate.edu (K.L.M.); 2 MRC Protein Phosphorylation and Ubiquitylation Unit, School of Life Sciences, University of Dundee, Dundee DD1 5EH, UK; i.ganley@dundee.ac.uk

**Keywords:** mTOR-independent autophagy, apoptosis, coibamide A, apratoxin A, ER stress, Sec61, BiP, GRP78

## Abstract

Our understanding of autophagy and lysosomal function has been greatly enhanced by the discovery of natural product structures that can serve as chemical probes to reveal new patterns of signal transduction in cells. Coibamide A is a cytotoxic marine natural product that induces mTOR-independent autophagy as an adaptive stress response that precedes cell death. Autophagy-related (ATG) protein 5 (ATG5) is required for coibamide-induced autophagy but not required for coibamide-induced apoptosis. Using wild-type and autophagy-deficient mouse embryonic fibroblasts (MEFs) we demonstrate that coibamide-induced toxicity is delayed in ATG5^−/−^ cells relative to ATG5^+/+^ cells. Time-dependent changes in annexin V staining, membrane integrity, metabolic capacity and caspase activation indicated that MEFs with a functional autophagy pathway are more sensitive to coibamide A. This pattern could be distinguished from autophagy modulators that induce acute ER stress (thapsigargin, tunicamycin), ATP depletion (oligomycin A) or mTORC1 inhibition (rapamycin), but was shared with the Sec61 inhibitor apratoxin A. Coibamide- or apratoxin-induced cell stress was further distinguished from the action of thapsigargin by a pattern of early LC3-II accumulation in the absence of CHOP or BiP expression. Time-dependent changes in ATG5-ATG12, PARP1 and caspase-3 expression patterns were consistent with the conversion of ATG5 to a pro-death signal in response to both compounds.

## 1. Introduction

Although macroautophagy (hereafter abbreviated to autophagy) and apoptosis are most conveniently studied as two distinct signal transduction pathways, the capacity for cross-talk between these processes has been appreciated for many years [1,2,3,4]. Autophagy can be induced beyond normal physiological levels as an early adaptive response to internal or external stress as cells attempt to maintain homeostasis through the “self-eating” of proteins and organelles [2]. In this survival mode, such as that triggered by starvation or exposure to an exogenous chemical, autophagy is favored and apoptosis signaling is inhibited [5]. However, if internal or environmental stress is sustained or cells fail to adapt, autophagy signaling can be suppressed or actively used by the cell to favor death signaling. These cell fate decisions can proceed efficiently through the use of intracellular signals that serve dual functions, in that autophagy-related (ATG) protein complexes regulate apoptosis and apoptotic proteins regulate autophagy [1,4]. The dynamic and context-specific nature of autophagy represents a current challenge to the development of autophagy modulators as therapeutic agents for the treatment or prevention of human disease and thus a better understanding of pro-survival versus pro-death roles of autophagy in cell fate is needed [6]. This is especially important in cancer where the function of autophagy changes over time at different stages of the disease [7].

With respect to pharmacological manipulation of autophagy, natural macrocyclic peptides and polyketides represent an important source of molecular structures with the potential to bind complex intracellular targets and reveal new aspects of cell signaling [8,9]. The utility of these molecules is demonstrated by the fact that some well-characterized macrocycles are already widely used as chemical probes for autophagy research [10]. For example, bafilomycin A1 was one of several bafilomycin structures originally isolated from *Streptomyces griseus* strains, that is now routinely used as a tool compound to assess autophagic flux [10,11]. The binding target of bafilomycin A1 is vacuolar (H^+^)-ATPase (V-ATPase), a hetero-oligomeric proton pump that is critical for autophagosome-lysosomal fusion [12,13,14,15,16]. Bafilomycin A1 can be used as a pharmacological inhibitor to block autophagosome-lysosomal fusion, and therefore autophagosomal degradation, in cultured cells [10]. Many more natural products are known to reliably modulate autophagy signaling by indirect mechanisms through binding to a specific regulatory target that lies outside the main autophagy pathway [17]. The macrocyclic polyketide rapamycin (sirolimus), originally from *Streptomyces hygroscopicus*, is perhaps the most famous natural product inducer of autophagy [10,18]. Through direct binding to FK506 binding protein 12, the rapamycin-FK506 complex can subsequently bind, and inhibit the function of, the serine/threonine kinase mechanistic Target of Rapamycin (mTOR) to induce autophagy [19,20]. The utility of natural products to probe very specific aspects of cell signaling is demonstrated by the fact that mTOR forms two distinct protein complexes in mammalian cells but these complexes display differential sensitivity to rapamycin [20]. Acute rapamycin treatment inhibits the activity of mTOR complex 1 (mTORC1), whereas mTOR complex 2 (mTORC2) is generally considered rapamycin-insensitive or inhibited only after long exposures in some cell types [20,21,22].

**Figure 1:**
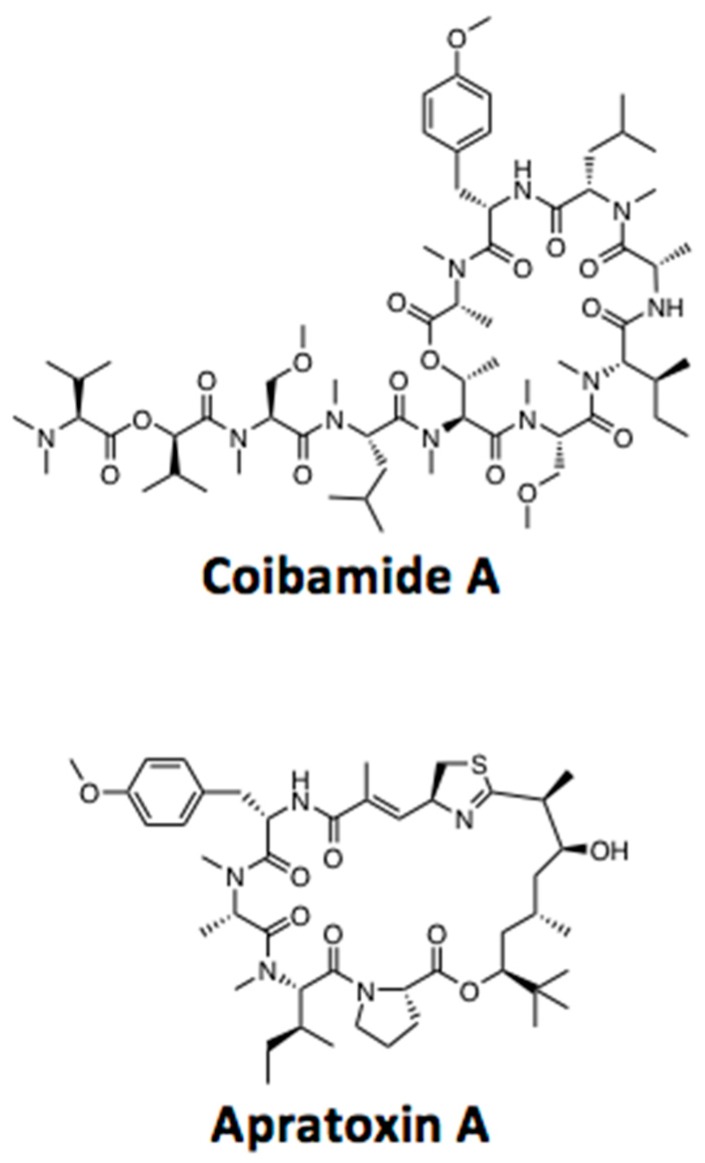
Chemical structures of coibamide A and apratoxin A.

In our studies of the chemistry and biology of the marine natural product coibamide A (Figure 1), we previously noted that this highly *N*-methylated lariat depsipeptide induces autophagy in cultured human cells and mouse fibroblasts within an hour of exposure [23]. This autophagy response displayed specificity, in that it was not due to a global block in the endocytic capacity of the cell, and occurred via a mechanism that is independent of mTOR kinase inhibition [23]. Autophagosome accumulation in response to coibamide A exposure was dependent on the presence of ATG protein 5 (ATG5), a highly conserved protein that is an essential component of the ubiquitin-like conjugation system, which sequentially drives formation of autophagosomes [24]. However, the presence of ATG5 was not required for cell death; coibamide A effectively induced caspase-dependent apoptosis in ATG5-null cells leading us to conclude that autophagy serves as an indirect stress response that persists in dying cells [23]. Given that coibamide A is capable of triggering apoptosis in the presence or absence of autophagy, we extended our analysis of wild-type and autophagy-deficient mouse embryonic fibroblasts (MEFs) to gain a better understanding of the role of coibamide-induced autophagy in cell fate. Genetically-modified and wild-type MEFs represented a convenient, non-cancer cell system for these studies as coibamide-induced cell death in human cancer cells is context dependent, and can proceed via apoptosis or alternate death pathways [23]. In the present study, the pharmacological action of coibamide A in MEFs was compared to that of other compounds for which the mechanism of action is known. We find evidence of a functional relationship between autophagy and apoptosis following coibamide A exposure, and show that the marine cyanobacterial natural product apratoxin A (Figure 1) produces the same pattern of responses in cells.

## 2. Results

### 2.1. Wild-Type and ATG5-Null MEFs Show Differential Sensitivity to Coibamide A

**Figure 2:**
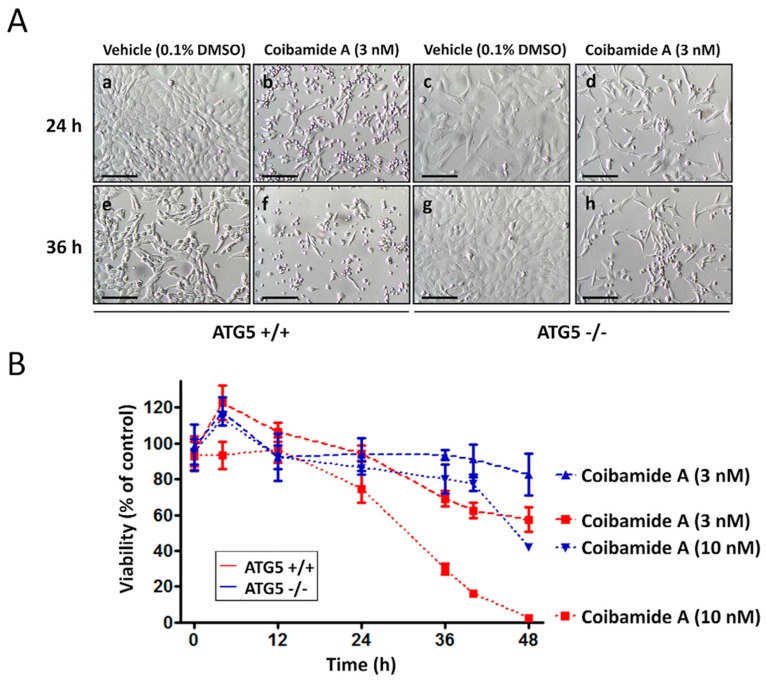
ATG5^+/+^ cells are more vulnerable to coibamide A than ATG5^−/−^ cells. (**A**) Morphological evaluation of wild-type and ATG5-null mouse embryonic fibroblasts (MEFs) in the presence of coibamide A (3 nM) or vehicle (0.1% DMSO). MEFs were treated as indicated for 24 h (panels a–d) or 36 h (panels e–h) and observed using light microscopy. Scale bar = 500 µm. (**B**) Trypan blue exclusion test of cell viability in wild-type and ATG5-null MEFs. Cells were seeded at 3000 cells/well and collected up to 48 h after treatment. Trypan Blue exclusion profiles represent mean cell counts ± SE of a time course performed in triplicate with the viability of vehicle-treated cells defined as 100%.

Using standard end-point viability assays we previously showed that autophagy is not needed for coibamide-induced cell death. Exposure to coibamide A (100 nM) resulted in caspase-dependent apoptosis in both wild-type cells and those lacking a functional autophagy pathway [23]. However, during routine examination of treated wild-type and ATG5-null MEFs by phase-contrast microscopy, morphological changes in the wild-type cultures were consistently detected first. To pursue this observation, we treated wild-type and ATG5-null MEFs with coibamide A or vehicle (0.1% DMSO) and assessed changes in cell morphology and viability over time. By 24 h more wild-type than ATG5-null MEFs showed characteristic signs of cell rounding and detachment from culture plates when exposed to low nanomolar concentrations of coibamide A (Figure 2A). This difference in response to coibamide A (3 nM) was more evident at 36 h, with very few wild-type MEFs showing normal morphology relative to ATG5-null MEFs (Figure 2A). When cell viability was quantified using a basic Trypan blue exclusion assay, it was apparent that the rate of cell death was accelerated in MEFs with a functional autophagy pathway (Figure 2B). Although coibamide A induced time- and concentration-dependent losses in membrane integrity in both wild-type and ATG5-null MEFs, relative to vehicle-treated control cells, wild-type cells were more sensitive to coibamide A than ATG5-null MEFs (Figure 2B).

**Figure 3:**
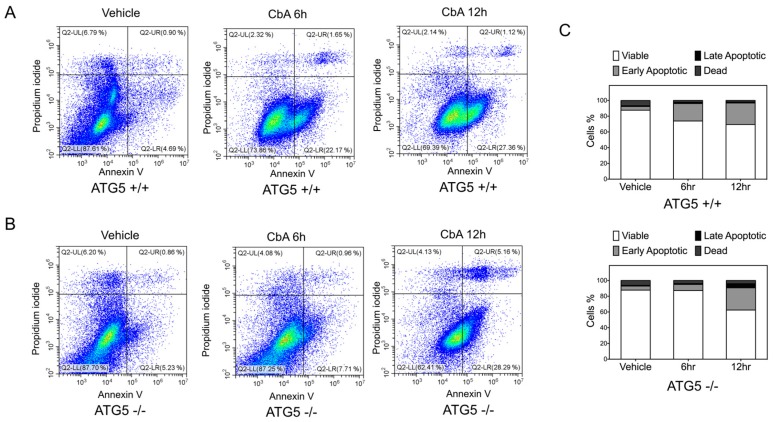
Analysis of Annexin V and propidium iodide (PI) staining of ATG5^+/+^ and ATG5^−/−^ cells following coibamide A treatment. (**A**) Wild-type and (**B**) ATG5-null mouse embryonic fibroblasts (MEFs) were labelled with annexin-V-FITC and PI to separate populations of viable (Lower Left quadrant: annexin V negative/PI negative), early apoptotic (Lower Right: annexin V positive/PI negative), late apoptotic (Upper Right: annexin V positive/PI positive) and dead/necrotic (Upper Left: annexin V negative/PI positive) cells using flow cytometry. Cells were treated with coibamide A (10 nM) or vehicle (0.1% DMSO) for up to 12 h before processing for FACS. Data was collected on a CytoFLEX Flow Cytometer using 1 µM staurosporine-treated cells as a positive control for induction of apoptosis. (**C**) Bar graph represents the percentage of viable, early-stage apoptotic, late-stage apoptotic and dead cells according to treatment. Figure is representative of comparisons made over 2–4 independent experiments.

FACS was used to quantify the potential for coibamide A to induce early apoptosis in each cell line as defined by expression of phosphatidylserine (PS) residues on the outer surface of the plasma membrane [25]. Wild-type and ATG5-null MEFs were treated with or without coibamide A (10 nM) for up to 12 h and then incubated with annexin-V conjugated to FITC and propidium iodide (PI) to distinguish early apoptosis versus secondary death signaling in response to treatment. Although the majority of cells were viable (annexin V negative/PI negative) under these conditions, a population of early apoptotic (annexin V positive/PI negative) cells was detected in wild-type cells after exposure times of 6 h and 12 h (Figure 3A). In contrast, ATG5-null MEFs remained viable, and indistinguishable from vehicle-treated control cells, at 6 h but acquired an early apoptotic signature by 12 h exposure to coibamide A (Figure 3B,C). To determine if the observed differences in response could be easily explained by inherent differences in cellular proliferation rate, we used real time impedance-based monitoring of cell spreading and growth, and also calculated the average doubling time of each cell line. These studies revealed no clear differences between the untreated wild type and knockout MEFs. Using seeding densities and culture conditions that were comparable to those used in our cell-based assays we found the adhesion and lag phase of both cell types to be similar (Figure S1A). In the period of logarithmic growth, corresponding to the time frame of coibamide A exposure, wild-type MEFs tended to proliferate more slowly than ATG5-null MEFs (Figure S1A) but this did not translate to a statistically significant difference in the average doubling time of each cell type (Figure S1B).

**Figure 4:**
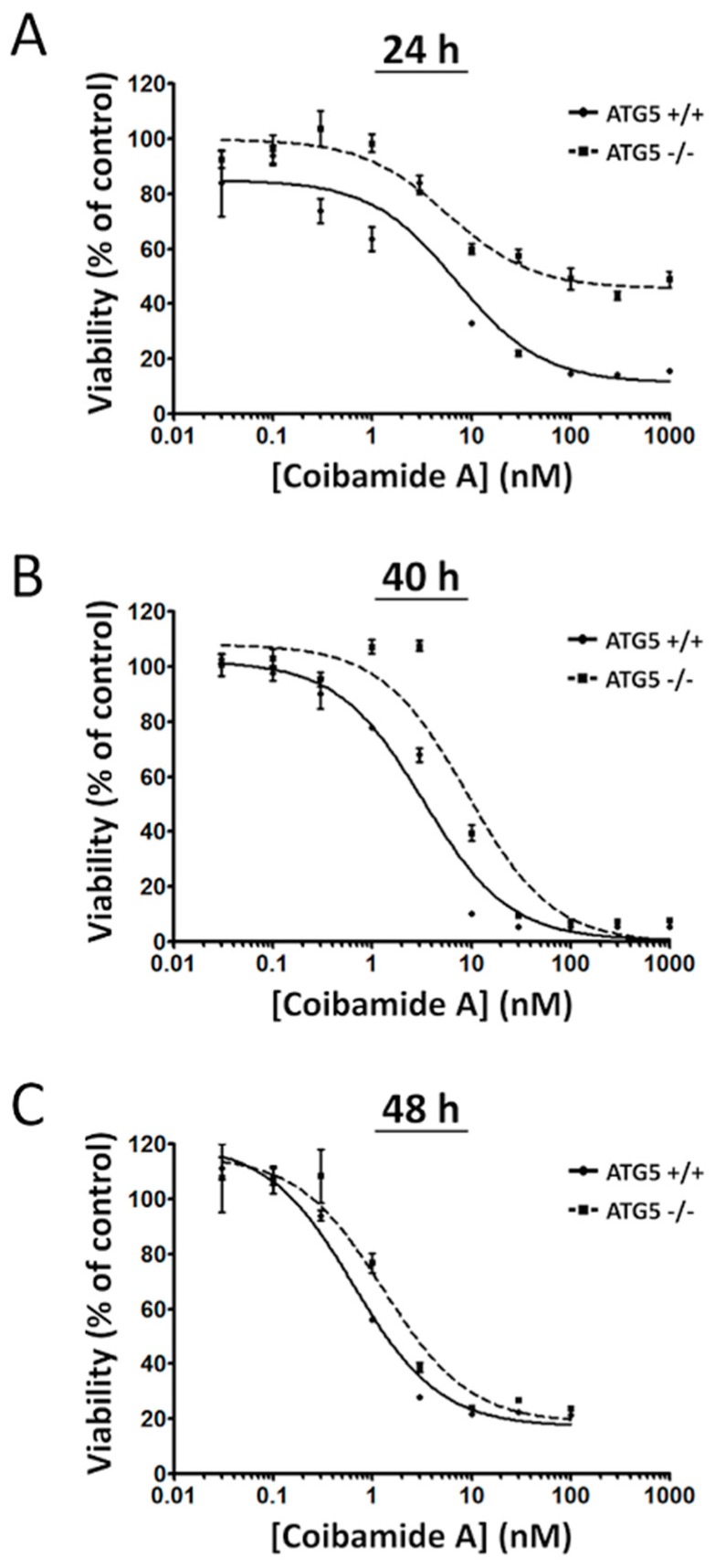
Coibamide-induced toxicity is delayed in ATG5^−/−^ cells. Time- and concentration-dependent changes in the viability of wild-type and ATG5-null mouse embryonic fibroblasts (MEFs) after exposure to coibamide A. Cells were exposed to increasing concentrations of coibamide A (0.3 nM to 1 µM) for (**A**) 24 h, (**B**) 40 h, or (**C**) 48 h. Cell viability was determined at each end-point with a WST-8 proliferation/cytotoxicity assay with the viability of vehicle-treated cells defined as 100%. Data points show mean viability ± SE (*n* = 3 wells per treatment) from a representative comparison that was repeated in three independent experiments.

We next treated wild-type and ATG5-null MEFs with increasing concentrations of coibamide A (0.03 nM to 1 µM) or vehicle (0.1% DMSO) and analyzed cell viability at three different end-points (24 h, 40 h, and 48 h). Using a WST-8 assay, which detects a loss in the metabolic capacity of cells as the read-out of cell viability, clear differences were observed in the sensitivity of wild-type versus ATG5-null MEFs. When assays were terminated at 24 h, coibamide A showed limited cytotoxic efficacy against ATG5-null MEFs (Figure 4A). If exposure times were extended to 40 h (Figure 4B) or 48 h (Figure 4C), the efficacy of coibamide A was enhanced against ATG5-null MEFs, however concentration-response curves for wild-type cells consistently fell to the left of ATG5-null MEFs (Figure 4A–C). Nonlinear regression analysis of these cell viability data revealed slight increases in the apparent potency of coibamide A against wild-type cells versus ATG5-null MEFs, but differences were not statistically significant by 48 h as anticipated from earlier studies [23].

**Figure 5:**
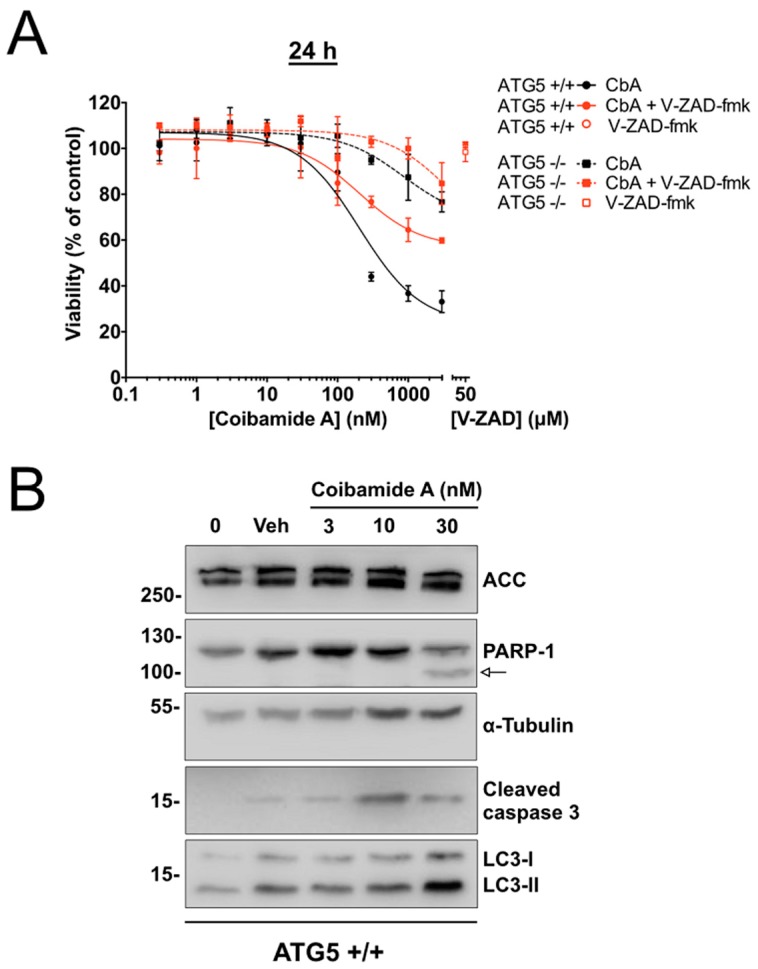
The pan caspase inhibitor V-ZAD-fmk inhibits coibamide-induced cytotoxicity in MEFs. (**A**) Cytoprotective effect of V-ZAD-fmk on both wild-type and ATG5-null mouse embryonic fibroblasts (MEFs) treated with coibamide A. Cells were exposed to increasing concentrations of coibamide A (0.3 nM to 3 µM), with or without V-ZAD-fmk (50 µM), and the viability was determined with a WST-8 proliferation/cytotoxicity assay at 24 h. The viability of vehicle-treated cells was defined as 100%. Data points show mean viability ± SE (*n* = 3 wells per treatment) from a representative comparison that was repeated in three independent experiments. (**B**) Expression of endogenous biomarkers of autophagy and caspase-dependent apoptosis in wild-type MEFs at 24 h. Immunoblot analysis of: poly [ADP-ribose] polymerase 1 (PARP-1), cleaved caspase-3 and LC3-I/II relative to alpha-tubulin and acetyl-CoA carboxylase (ACC), in cells treated with, or without (0), vehicle (0.1% DMSO) or coibamide A (3–30 nM) for 24 h. Whole cell lysates were probed with appropriate primary antibodies as indicated. Cleavage product of PARP-1 is denoted by an arrow. Each series of blots is representative of patterns that were observed in at least three independent experiments.

The viability of both cell types was enhanced, however, when cells were co-treated with coibamide A and the pan caspase inhibitor Z-VAD-fmk (50 µM). For these studies assays were terminated at 24 h to better distinguish responses in wild-type versus ATG5-null cells. This analysis resulted in concentration-response relationships that were shifted in co-treated wild-type and ATG5-null MEFs relative to cells treated only with coibamide A (Figure 5A). Z-VAD-fmk alone produced no change in the viability of either cell line, whereas over 50% of co-treated wild-type cells were still viable at 24 h in the presence of high concentrations of coibamide A (1–3 µM) and Z-VAD-fmk (Figure 5A). Immunoblot analysis of whole-cell lysates harvested from adherent wild-type MEFs treated with coibamide A (3–30 nM), showed concentration-dependent accumulation of the lipidated form of ATG8/LC3, LC3-II, a marker of the autophagosomal membrane [10], and the proteolytic processed forms of PARP1 and caspase-3 [26] (Figure 5B). This biochemical evidence of apoptosis signaling in coibamide-stressed cells coupled with the cytoprotective effect of Z-VAD-fmk, regardless of ATG5 status, is consistent with caspase-dependent apoptosis as a primary death mechanism in MEFs in response to coibamide A.

**Figure 6:**
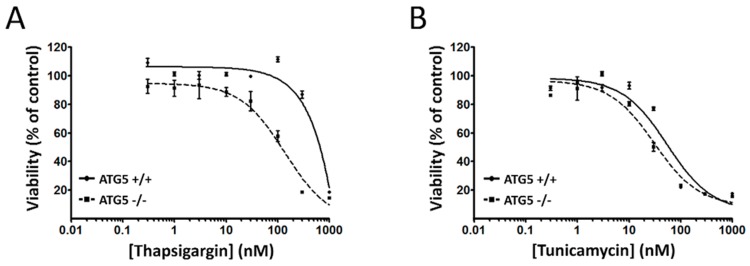

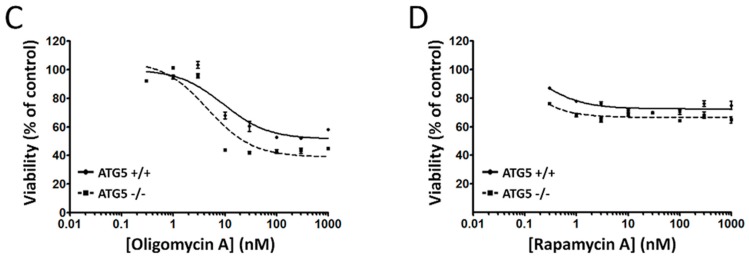
Comparison of ATG5^+/+^ and ATG5^−/−^ cell viability in response to known modulators of autophagy. Concentration-dependent changes in the viability of wild-type and ATG5-null mouse embryonic fibroblasts (MEFs) after exposure to (**A**) thapsigargin, (**B**) tunicamycin, (**C**) oligomycin A, and (**D**) rapamycin A. ATG5^+/+^ and ATG5^−/−^ cells were exposed, in parallel, to increasing concentrations of each compound (0.3 nM to 1 µM) for 48 h. Cell viability was determined by a WST-8 proliferation/cytotoxicity assay, with the viability of vehicle-treated cells defined as 100%. Data points show mean viability ± SE (*n* = 3 wells per treatment) from a representative comparison that was repeated in three independent experiments.

To understand if the absence of ATG5 confers the same pattern of differential sensitivity to other compounds, the activity of coibamide A was tested relative to several reference compounds that are known to influence autophagy via indirect mechanisms. When the viability of wild-type and ATG5-null MEFs was tested in response to increasing concentrations of pharmacological inducers of ER stress (thapsigargin and tunicamycin), an inhibitor of ATP synthase (oligomycin A) or rapamycin, none of the compounds gave a pattern that matched that of coibamide A (Figure 6A–D). The viability and/or growth characteristics of wild-type and knockout cells was changed in response to increasing concentrations of all four reference compounds, however, in each case the ATG5-null MEFs were either more sensitive, or as sensitive, as the wild-type cells in this assay (Figure 6). Taken together, these results demonstrate that autophagy-competent cells are more vulnerable to coibamide A-induced apoptosis than autophagy-deficient MEFs in a pattern that does not generalize to several other well characterized modulators of autophagy. 

### 2.2. Coibamide A-Induced Autophagy Is Not Triggered by Acute ER Stress

**Figure 7:**
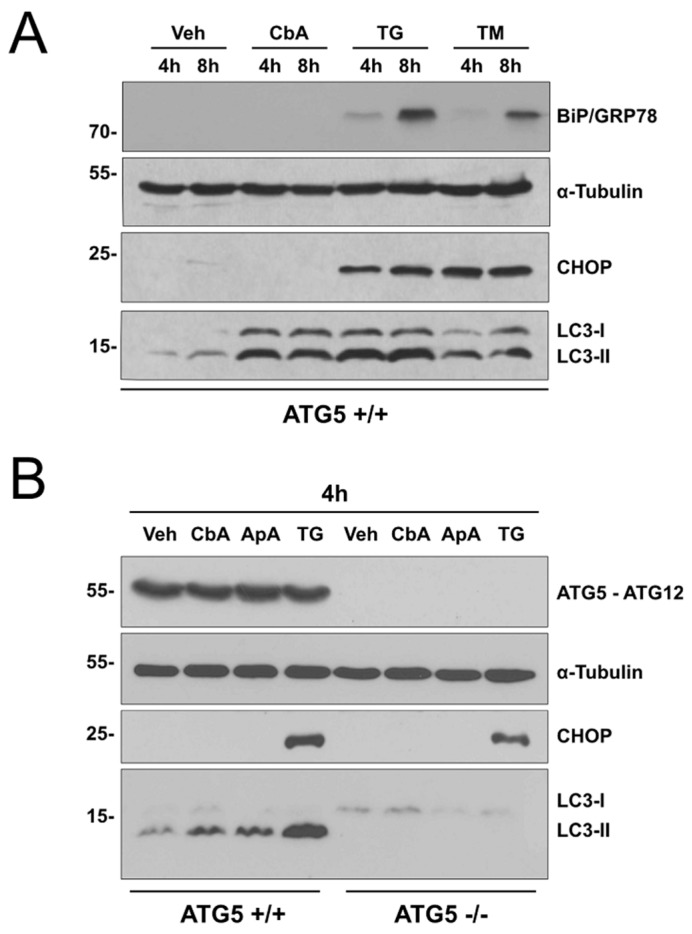
Coibamide A-induced autophagy is not triggered by acute ER stress. Expression analysis of endogenous LC3-II, CHOP, ATG5, and BiP in wild-type and ATG5-null mouse embryonic fibroblasts (MEFs) in response to short-term coibamide A treatment. Immunoblot analysis of: (**A**) LC3-I/II, BiP and CHOP relative to alpha-tubulin after treatment with vehicle (0.1% DMSO), coibamide A (30 nM), thapsigargin (10 μM), and tunicamycin (20 μg/mL) for 4 h and 8 h and, (**B**) LC3 I/II, ATG5, ATG12, and CHOP relative to alpha-tubulin after treatment with vehicle (0.1% DMSO), coibamide A (30 nM), apratoxin A (30 nM), and thapsigargin (10 µM) for 4 h. Whole cell lysates were probed with primary antibodies as indicated; note that ATG5 is detected in the context of the covalent ATG5-ATG12 complex. Each series of blots is representative of patterns that were observed in at least three independent experiments.

As ATG5-null and wild-type MEFs showed contrasting patterns of sensitivity to thapsigargin (Figure 6A) and coibamide A (Figure 4) we tested the ability of coibamide A to induce a typical ER stress response. For these studies, wild-type MEFs were treated for 4 and 8 h with fixed concentrations of coibamide A (30 nM), thapsigargin (10 μM), tunicamycin (20 μg/mL), or vehicle (0.1% DMSO) and whole cell lysates collected for immunoblot analysis. All three compounds induced accumulation of LC3-II relative to control, however, coibamide A failed to induce expression of common ER-stress biomarkers: binding immunoglobulin protein (BiP), also known as glucose-regulated protein of 78 kDa (GRP78), or CAAT-enhancer-binding homologous protein (CHOP) [27,28] (Figure 5A). This lack of BiP and CHOP immunoreactivity was in contrast to thapsigargin- and tunicamycin-treated cells, which showed characteristic increases in both BiP and CHOP expression in this time frame (Figure 7A). We next compared the action of coibamide A to the marine natural product apratoxin A. Apratoxin A is the first of a series of macrocyclic depsipeptides originally isolated by Luesch and co-workers from cyanobacteria growing in Apra Harbor, Guam [29]. In previous testing against normal human vascular endothelial cells (HUVECs), apratoxin A induced morphological and biochemical changes that, to date, most closely match the action of coibamide A [30]. For these studies, wild-type and ATG5-null MEFs were treated for 4 h with a single concentration of coibamide A (30 nM), apratoxin A (30 nM), thapsigargin (10 μM), or vehicle (0.1% DMSO). Immunoblot analysis revealed accumulation of LC3-II in wild-type cells treated with all three compounds, in a pattern consistent with the requirement of ATG5 protein for LC3 conversion, however, expression of CHOP was observed only in response to thapsigargin and was induced in both wild-type and ATG5-null cells (Figure 7B).

### 2.3. Comparison of Coibamide- and Apratoxin-Induced Cell Death

**Figure 8:**
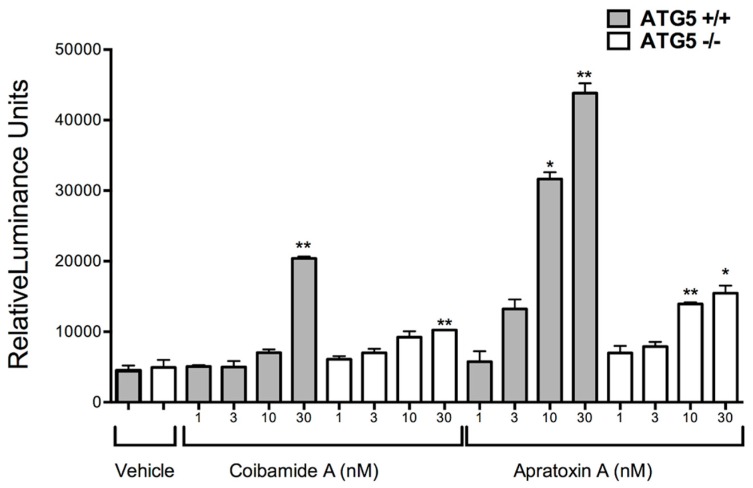
Caspase-3,7 activation is delayed in ATG5^−/−^ cells exposed to coibamide A or apratoxin A. Concentration-dependent analysis of coibamide- or apratoxin-induced caspase activity in wild-type and ATG5-null mouse embryonic fibroblasts (MEFs). Cells were exposed to increasing concentrations of coibamide A (1 nM to 30 nM), apratoxin A (1 nM to 30 nM), or vehicle (0.1% DMSO) for 24 h. Bars represent mean luminescence activity expressed as Relative Light Units ± SE (*n* = 3 wells per treatment) from three independent comparisons. Statistical significance of activity in treated MEFs relative to control is indicated as * *p* < 0.05 and ** *p* < 0.01.

To investigate the possibility that autophagy-competent cells are more sensitive to apratoxin A, the ability of both compounds to induce apoptosis was compared. Cells were treated in parallel over a range of coibamide A or apratoxin A concentrations (1–30 nM) and tested for activity of the major downstream effector caspases-3 and -7. Although apratoxin A was the more potent activator of caspase-3,7, the two compounds displayed similar patterns of activity in both cell lines (Figure 8). Apratoxin A and coibamide A induced concentration-dependent activation of caspases-3,7 with higher activity in wild-type MEFs relative to ATG5-null cells at 24 h (Figure 8). These results suggest that apratoxin A and coibamide A share a common pattern of cell death signaling, at least in fibroblasts. Both compounds: (1) induce autophagy in the absence of acute ER stress and, (2) induce caspase-dependent apoptosis in the absence of autophagy. Moreover, the absence of a functional autophagy pathway confers the same survival advantage in response to both compounds.

The concentration-response relationship for caspase-3,7 activation by both natural products (Figure 8) was used to inform an assessment of the stability of the ATG5-ATG12 protein complex relative to biomarkers of autophagy and caspase-dependent apoptosis. Although essential for autophagosome formation, ATG5 can undergo proteolytic cleavage to liberate an N-terminal, unconjugated ATG5 fragment that functions as a pro-apoptotic signal [31]. For these studies, cells were treated with coibamide A (30 nM), apratoxin A (30 nM), or vehicle (0.1% DMSO) and cell lysates were collected for up to 30 h for immunoblot analysis of ATG5, SQSTM1/p62, LC3, PARP1, and caspase-3. SQSTM1/p62 is an LC3 binding protein that is destroyed during autophagy and can be used as an indirect measure of autophagy in combination with other biomarkers [2,10]. As we had previously noted that coibamide A induces a concentration- and time-dependent detachment of MEFs and human glioblastoma cells from cell culture dishes [23], we also collected any detaching cells (24–30 h) and analyzed this population separately. A statistically significant decrease in ATG5 expression (detected as loss of ATG5-ATG12) was observed after exposure to either coibamide A (30 h) or apratoxin A (18 to 30 h) relative to control cells treated with vehicle (0.1% DMSO) for 30 h (Figure 7A,B). Unfortunately, efforts to detect the specific N-terminal cleavage fragment of ATG5 were not successful due to high non-specific binding of the antibody in coibamide- or apratoxin-treated cell lysates (data not shown). 

**Figure 9:**
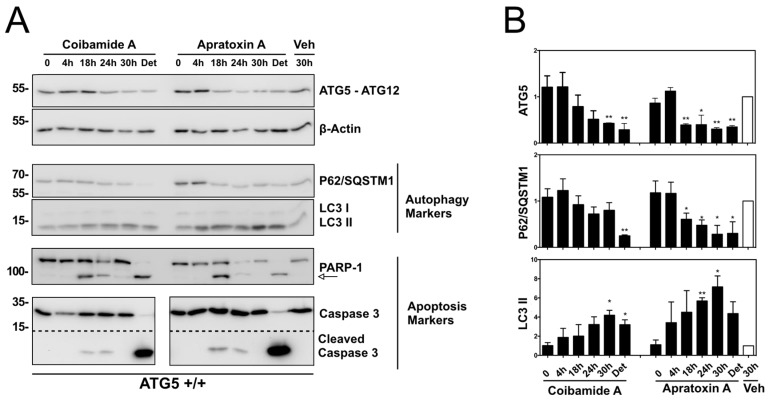
Time-dependent analysis of autophagy and apoptosis signals in ATG5^+/+^ cells exposed to coibamide A or apratoxin A. Wild-type mouse embryonic fibroblasts (MEFs) were treated with, or without (0 unit), coibamide A (30 nM), apratoxin A (30 nM), or vehicle (Veh; 0.1% DMSO) for up to 30 h. Whole cell lysates were collected from adherent and detached (Det) cells (24 and 30 h). (**A**) Immunoblot analysis of ATG5 (detected in the context of the covalent ATG5-ATG12 complex), P62/SQSTM1, LC3, PARP1, caspase 3 expression relative to beta-actin. (**B**) Quantitation of immunoblot data shown in (**A**). Bars represent intensity of bands normalized to beta-actin, relative to vehicle-treated cells (open bars) in three independent experiments. Statistical significance is indicated as * *p* < 0.05 and ** *p* < 0.01.

These lysates did, however, reveal a steady decrease in expression of SQSTM1/p62 that was statistically significant for apratoxin-treated cells, and a general increase in LC3-II levels over time (Figure 9A,B). With respect to apoptosis, proteolytic processed forms of PARP1 and caspase-3 were detected by 18 h of exposure to either coibamide A or apratoxin A, consistent with the timing of caspase-3,7 activation (Figure 8), whereas lysates harvested at earlier time points (0 and 4 h) or after 30 h of vehicle treatment expressed only the unprocessed forms of PARP1 and caspase-3 (Figure 7A). Particularly strong immunoreactivity corresponding to the cleaved form of caspase-3 was observed in detached populations of both coibamide- and apratoxin-treated cells, accompanied by a loss of the inactive 32 kDa form of this enzyme relative to all adherent cells. Similarly, unprocessed PARP1 was barely detectable in the detached cell lysates that also showed low levels of ATG5, low SQSTM1/p62, and increased LC3-II relative to the adherent vehicle-treated cells (Figure 9A). 

### 2.4. Partial Rescue of the Wild-Type Phenotype by Expression of ATG5 in ATG5-Null MEFs

**Figure 10:**
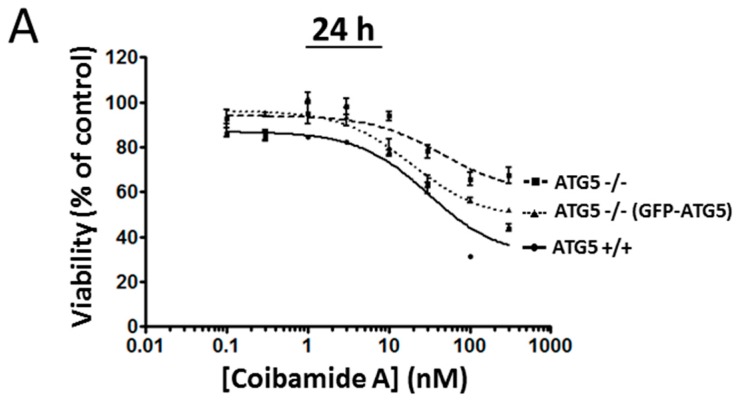

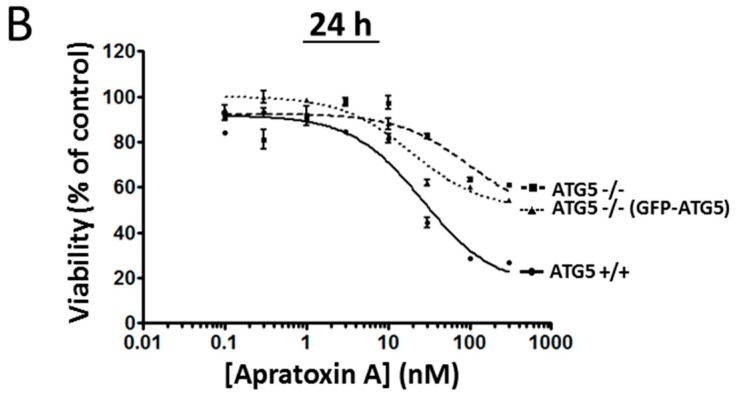
Partial rescue of the wild-type phenotype after re-expression of ATG5 in ATG5^−/−^ cells. Concentration-dependent changes in the viability of wild-type, ATG5-null, and ATG5-null re-expressing ATG5 (ATG5^−/−^ (GFP-ATG5)) in mouse embryonic fibroblasts (MEFs), in response to (**A**) coibamide A or, (**B**) apratoxin A exposure. Cells were exposed to increasing concentrations of coibamide A (0.1 nM to 300 nM), apratoxin A (0.1 nM to 300 nM), or vehicle (0.1% DMSO) for 24 h. Cell viability was determined in a WST-8 proliferation/cytotoxicity assay, with the viability of vehicle-treated cells defined as 100%. Data points show mean viability ± SE (*n* = 3 wells per treatment) from a representative comparison that was repeated in three independent experiments.

The role of ATG5 in coibamide A- and apratoxin A-induced cell death was investigated further using a stable cell line, where ATG5-null MEFs had been transduced to re-express ATG5 in the null background [32], and then purified by clonal selection (Figure S2). All three cell lines (wild-type, ATG5-null and ATG5^−/−^ (GFP-ATG5)) were treated with vehicle (0.1% DMSO) or increasing concentrations of either coibamide A or apratoxin A for 24 h to better distinguish responses in wild-type versus ATG5-null cells (Figure 4A). In these assays, we observed the same shift in the sensitivity of ATG5^−/−^ cells expressing ATG5 to both coibamide A (Figure 10A) and apratoxin A (Figure 10B). These cells responded with intermediate sensitivity to both coibamide A and apratoxin A, relative to wild-type and ATG5-null MEFs, suggesting that re-expression of ATG5 resulted in a partial rescue of the wild-type phenotype. Taken together, these results indicate that ATG5 has a functional influence on coibamide A- and apratoxin A-induced cell death. 

## 3. Discussion

The cyanobacterial secondary metabolites coibamide A (The stereochemistry of natural coibamide A was subsequently revised by Yao and coauthors. Yao, G., Pan, Z., Wu, C., Wang, W., Fang, L., Su, W. (2015) Efficient Synthesis and Stereochemical Revision of Coibamide A. J. Am. Chem. Soc. 137, 13,488–13,491) and apratoxin A were discovered independently as new, structurally-unrelated cyclic depsipeptides with striking anti-proliferative and cytotoxic activity against human cancer cells [29,33]. We previously determined that both compounds induce macroautophagy within hours of exposure as part of a protective response to cytotoxic stress [23,30]. In an analysis of LC3-II turnover we observed enhanced autophagic flux in the early stages of exposure to coibamide A and, with longer exposures, a tendency for dying cells to accumulate LC3-II [10,23]. In the present study, we provide insight into the functional role of autophagy in mammalian cells after exposure to these complex natural products. Coibamide A and apratoxin A induced autophagy before the onset of caspase-dependent apoptosis, consistent with a well-recognized pattern in which cells undergo autophagy and apoptosis in sequence [2]. Moreover, evidence of caspase-dependent apoptosis was temporally correlated with a decline in ATG5 expression, suggesting that ATG5 plays an active role in the switch from survival to pro-death signaling in response to a lethal concentration of either compound. Although coibamide A and apratoxin A also induced cell death in ATG5-null cells, side-by-side comparisons in multiple assays revealed a delayed response relative to cells with a functional autophagy pathway. The pattern of responses evoked by coibamide A and apratoxin A in wild-type and autophagy-deficient fibroblasts could be distinguished from autophagy modulation as a result of mTORC1 inhibition (rapamycin), ER stress (thapsigargin and tunicamycin) or ATP depletion (oligomycin A). As the primary mechanism of action of coibamide A and apratoxin A becomes clearer, these data demonstrate the potential utility of these cyclic depsipeptide structures as future molecular probes to study the relationship between a distinct type of stress-induced autophagy and the conversion of ATG5 to a pro-apoptosis signal.

Although the binding target of coibamide A has not yet been determined, preliminary studies suggest that coibamide A may be an inhibitor of the ER secretory pathway with some similarity to apratoxin A [30]. Apratoxin A, which has been studied more extensively, is known to act by inhibiting cotranslational translocation of a subset of proteins that are sorted to the secretory compartment of mammalian cells, including receptor tyrosine kinases, growth factors and cytokines [34,35]. This action occurs as a result of direct binding of apratoxin A to the alpha, or channel-forming, subunit of the Sec61 protein translocation channel located at the entrance to the ER secretory pathway [36,37]. Inhibition of Sec61 client proteins by apratoxin A leads to a pattern of cellular consequences, some of which appear to be shared with coibamide A, including induction of mTOR-independent autophagy and a cell cycle phase-specific block in G_1_ that precedes cell death [23,30,33,34]. In the present study, loss of viability in response to coibamide A and apratoxin A was delayed in ATG5-null MEFs, whereas cells treated with pharmacologic inducers of ER or mitochondrial stress showed the opposite response. ATG5-null, rather than wild-type, cells were generally more sensitive to tunicamycin, thapsigargin, and oligomycin A. The enhanced sensitivity of ATG5-null cells to tunicamycin and thapsigargin is consistent with previous analyses of ER-stress-dependent cell death in these cells. Ogata and coworkers originally showed that ATG5-null cells have increased vulnerability to ER stress, in keeping with a well-established protective role for autophagy in response to ER stress [38,39]. A second feature that distinguished the action of coibamide A and apratoxin A from that of pharmacologic inducers of ER stress, was a lack of CHOP and BiP expression in response to either cyclic depsipeptide despite evidence of LC3-II accumulation and apoptosis signaling. In contrast, we found tunicamycin and thapsigargin to be strong inducers of CHOP and BiP, as anticipated from early characterization of these compounds [40,41,42]. Early studies by the Luesch laboratory also distinguished the action of apratoxin A from tunicamycin on the basis of their ability to produce opposing effects on BiP expression [35]. This lack of BiP and CHOP induction in response to coibamide A or apratoxin A, is instead more consistent with the lack of conventional ER stress biomarkers noted after exposure to the Sec61 inhibitor mycolactone [43]. Mycolactone, is a natural product macrolide, produced as a virulence factor by *Mycobacterium ulcerans*, which binds Sec61 alpha, but does not induce BiP expression, phosphorylation of PERK or cause IRE-dependent splicing of XBP-1 in RAW264.7 macrophage cells [43,44]. 

ATG5 functions in the context of a cytosolic ATG5-ATG12 protein complex that is essential for autophagosome formation [45]. The total knockout of ATG5 in mice results in animals that die hours after birth due to a failure to adapt to the normal state of nutrient depletion that occurs in the early phase of postnatal life [46]. ATG5 is also an example of a protein that has functions outside the autophagy pathway and is recognized as a central node for conversion of a cytoprotective autophagy response to pro-death signaling via multiple mechanisms [2]. Cleavage of ATG5 by calpain inactivates autophagy signaling and produces a truncated N-terminal fragment of the ATG5 protein that translocates to the mitochondria to induce apoptosis [31,47]. Although we were unable to detect this transient N-terminal fragment in lysates harvested from dying cells, immunoreactivity corresponding to the full length ATG5 protein complex declined in response to coibamide A or apratoxin A. The importance of ATG5 was also demonstrated by the fact that re-expression of ATG5, in the ATG5-null background, partially restored the sensitivity of fibroblasts to either coibamide A or apratoxin A. Similar pro-death roles for ATG5 have been described in a number of other experimental settings when ATG5 levels were either enhanced or suppressed and compared to wild-type cells. In earlier studies by Yousefi and co-workers, silencing of ATG5 in cultured human HeLa cervical or MDA-MA-231 triple negative breast cancer cells, with short interfering RNA (siRNA), rendered these cells more resistant to staurosporine or doxorubicin for several days and delayed cell death [31]. ATG5 has also been shown to be strongly induced by the DNA-damaging agents etopside and cisplatin, and under these conditions is translocated to the nucleus to cause ATG5-dependent mitotic catastrophe and early cell death by apoptosis [48]. Furthermore, after tissue-specific knockout of ATG5 in the acinar cells of the salivary gland, ATG5-null acinar cells showed delayed apoptosis and were more resistant to H_2_O_2_-induced stress relative to cells with a functional autophagy pathway, which showed early apoptosis and senescent phenotypes [49].

In side-by-side comparisons of untreated, non-synchronized cells, ATG-null cells showed no significant difference in average doubling time. As there is potential for cross talk between autophagy signaling and cell cycle progression [50], it is possible, however, that wild-type and ATG5-null cells responded to the anti-proliferative stress of coibamide A or apratoxin A exposure differently. Studies in non-synchronized cancer cells indicate that the potent anti-proliferative action of both natural products is due to induction of a phase-specific block in G_1_ of the cell cycle [30,33,34]. It is still unclear, however, if activation of autophagy is correlated with a specific phase of the cell cycle [47]. A previous analysis of different pharmacologic inducers of autophagy found autophagy activation to be associated with G_1_ and S phases [50], whereas other studies have shown no specific link [51]. A clear role for autophagy, and for ATG5 specifically, has been demonstrated for the mechanism by which cells exit the cell cycle and become senescent [52], and thus it is assumed that other phases of the cell cycle were not impacted by the absence of ATG5 in our experiments although we did not test this directly. 

Previous studies have shown that a distinct form of autophagy-dependent, non-apoptotic cell death, termed autosis, can be induced by small synthetic peptides, which trigger death with morphological and biochemical characteristics that can be distinguished from apoptosis and other modes of cell death, such as necroptosis [53,54]. Given that wild-type and ATG5-null cells showed clear caspase-dependent apoptosis after exposure to coibamide A or apratoxin A it is unlikely that either of these cyclic depsipeptides are selective inducers of autosis. Further, cell death by autosis is characterized by several unique features including increased cell-substrate adhesion [54,55], whereas coibamide A and apratoxin A tend to show decreased cell-substrate adhesion leading to cell detachment (Figure 7 and [23]). The fungal heptadepsipeptide HUN-7293 was originally identified in a screen for potent inhibitors of cell adhesion before later characterization of natural and synthetic analogues revealed an ability of these molecules to target the Sec61 translocation channel [56,57,58]. Mycolactone has recently been reported to induce anoikis [59], a specific mode of cell death classified as detachment-induced apoptosis [26]. This finding raises the possibility that cytotoxic Sec61 inhibitors will induce cell detachment as a function of their ability to block biosynthesis of cell adhesion molecules. Further studies will be required to define the precise relationship between cell death and loss of cell-substrate adhesion in response to coibamide A as our previous studies indicate that coibamide-induced cell death can proceed via apoptosis or non-apoptotic pathways depending on the cell type. For example, U87-MG glioblastoma cells and Apaf-1-null MEFs could be induced to detach from culture dishes without any evidence of caspase-3 activation [23], and thus fail to meet the criteria for anoikis. 

In summary, coibamide A and apratoxin A appear to be examples of cytotoxic natural products that promote cross-signaling between ATG5-dependent autophagy and caspase-dependent apoptosis. These relatively new cytotoxins may also prove to be valuable tools for the study of alternate modes of regulated cell death in mammalian cells. These results indicate that mTOR-independent autophagy, without activation of common ER stress markers, may represent an early adaptation to changes in proteostasis after pharmacological inhibition of Sec61 function. Significantly, the absence of BiP/GRP78 and CHOP as biomarkers of acute cell stress potentially distinguishes autophagy stress triggered by cytotoxic Sec61 inhibitors, such as apratoxin A and mycolactone, from that caused by compounds that rapidly disrupt proteostasis within the ER, such as thapsigargin and tunicamycin. 

## 4. Materials and Methods

### 4.1. Chemicals, Reagents and Antibodies

Coibamide A was re-isolated from material collected by hand using SCUBA from Coiba National Park, Panama, and apratoxin A was isolated from a laboratory culture of a Red Sea strain of Moorea producens [33,60]. Rapamycin, thapsigargin, and tunicamycin were purchased from Sigma-Aldrich Corp. (St. Louis, MO, USA). Oligomycin A was from Santa Cruz Biotechnology Inc. (Santa Cruz, CA, USA) and Z-VAD-fmk from ApexBio (Houston, TX, USA). All compounds were reconstituted in 100% cell culture grade DMSO and stored at −20 °C until the day of treatment. The final concentration of DMSO was 0.1% for all studies. General laboratory reagents were from Sigma-Aldrich Corp. or VWR International (Radnor, PA, USA). Primary and secondary antibodies were commercial sources and used according to recommended protocols. Codes for primary antibodies from Cell Signaling Technology, Inc. (Danvers, MA, USA) were as follows: LC3A/B (#4108), ATG5 (D5F5U; #12994) as conjugated ATG5-ATG12, CHOP (#5554), BiP/GRP78 (#3177), acetyl-CoA carboxylase (#3676), GAPDH (#5174), alpha-tubulin (#2125), beta-actin (#4970S), caspase-3 (#9662S), and PARP-1 (#9532). Anti-SQSTM1/p62 (#ab91526) was from Abcam (Cambridge, MA, USA) and a second anti-ATG5 (N-term; #AP1812a) antibody was from Abgent, San Diego, CA, USA.

### 4.2. Mammalian Cell Culture

Wild-type and ATG5^−/−^ embryonic fibroblasts prepared from 13.5 day mouse embryos were a kind gift from Dr. Noboru Mizushima, Tokyo Medical and Dental University [46]. ATG5-null MEFs transduced to re-express ATG5 in the null background have been described and tested previously [32]. All MEFs were cultured in Dulbecco’s Modified Eagle’s Medium (DMEM; Mediatech Inc., Manassas, VA, USA), supplemented with 10% fetal bovine serum (FBS; HyClone, Logan, UT, USA), l-glutamine (2 mM), 100 I.U./mL penicillin, and 100 µg/mL streptomycin (1% penicillin/streptomycin; Mediatech Inc.).

### 4.3. Analysis of Cell Morphology, Viability and Caspase Activity

Cell morphology was examined and recorded using a Leica DM IL LED microscope fitted with a Leica DFC400 digital camera. Cell viability was assessed using a WST-8 proliferation/cytotoxicity assay (Cayman Chemical Company, Ann Arbor, MI, USA; #10010199) or a Trypan blue exclusion test, with the viability of vehicle-treated cells defined as 100% in all analyses. For WST-8 assays, MEFs were seeded into 96-well plates at a density of 2000 cells/well in 50 µL of medium. After 18 h, cells were treated with a range of concentrations of coibamide A (0.3 nM–1 µM), apratoxin A (0.3 nM–1 µM) or vehicle (0.1% DMSO) delivered in 50 µL of medium. In separate studies, cells were treated with or without coibamide A in the presence of Z-VAD-fmk (50 µM). For Trypan blue exclusion assays, MEFs were seeded at a density of 3000 cells/well prior to treatment with coibamide A (3 nM and 10 nM) or vehicle (0.1% DMSO). Cells were collected up to 48 h after treatment using Trypsin/EDTA (0.25%) and re-suspended in serum-free medium. Trypan blue reagent (Mediatech Inc., 25-900-Cl) was added to the cell suspension at a ratio of 1:1, and cells counted and scored (stained and unstained) by microscopy using a hemocytometer. For caspase activation assays, cells were seeded at a density of 2,000 cells/well into clear bottom white-walled plates (Greiner CellStar^®^ Kremsmünster, Austria) and caspase 3/7 activation assessed using a Caspase-Glo^®^ 3/7 luminescent assay (Promega, Fitchburg, WI, USA) according to the protocol for multi-well plate formats. 

### 4.4. Detection of Annexin V-FITC and Propidium Iodide by FACS

Wild-type and ATG5-null MEFs were exposed to coibamide A (10 nM) or vehicle (0.1% DMSO) for up to 12 h under standard culture conditions, washed in cold phosphate-buffered saline (PBS), and resuspended in annexin binding buffer (50 mM HEPES, 700 mM NaCl, 12.5 mM CaCl_2_, pH 7.4) before the addition of Annexin V-FITC (2.5 µL per 100 µL cells) and propidium iodide (0.02 µg/µL) (Invitrogen). Cells were incubated at room temperature for 15 min, washed in binding buffer and analyzed immediately by FACS using a CytoFLEX Flow Cytometer (Beckman Coulter Life Sciences, Brea, CA, USA). A number of 50,000 events were recorded and represented as density plots. MEFs treated with 1 µM of Staurosporine (Enzo Life Sciences Inc., Farmingdale, NY, USA) for 6 h was used as positive control for apoptosis. 

### 4.5. Immunoblot Analysis

At the end of treatment, plates were placed on ice and the cell monolayers rinsed with PBS, lysed and processed as described previously [23]. Briefly, cells were collected by gentle centrifugation, the cell pellet rinsed in PBS and then re-suspended in lysis buffer. Cell lysates were cleared by centrifugation at 16,000× *g* for 20 min at 4 °C and the protein concentration determined using the bicinchoninic acid (BCA) (Thermo Fisher Scientific Inc., Waltham, MA, USA). Cell lysates were adjusted for protein content and equal amounts of total protein were separated by SDS-PAGE and immobilized onto PVDF membranes. Membranes were then blocked in 5% (*w*/*v*) non-fat dry milk in 50 mM Tris-HCl, pH 7.4, 150 mM NaCl (TBS) plus 0.1% Tween-20 (TBS-Tween), and subsequently incubated for 16 h at 4 °C with the appropriate primary antibody in 5% (*w*/*v*) bovine serum albumin (BSA). The following day, membranes were washed in TBS-Tween (3 × 10 min) and incubated with the appropriate HRP-conjugated secondary antibody for 1 h at room temperature. Finally, membranes were washed in TBS-Tween (4 × 5 min), and proteins revealed using an enhanced chemiluminescence (ECL) reagent.

### 4.6. Data Analysis

Concentration-response relationships were analyzed using Graphpad Prism Software (Graphpad Software Inc., San Diego, CA, USA), and EC_50_ values derived using nonlinear regression analysis fit to a logistic equation. Statistical significance of cell viability was assessed using a one-way analysis of variance (ANOVA) followed by a student’s *t*-test to compare control and treatment groups. 

## Supplementary Materials

The following are available online at http://www.mdpi.com/1660-3397/16/3/77/s1, Figure S1: Comparison of cellular proliferation and doubling time in untreated wild-type, ATG5-nul MEFs. Figure S2: Analysis of ATG5 expression in wild-type, ATG5-null and ATG5-null mouse embryonic fibroblasts re-expressing GFP-ATG5.

## Abbreviations

The following abbreviations are used in this manuscript:

PVDF	Polyvinylidene difluoride
SDS-PAGE	sodium dodecyl sulfate-polyacrylamide gel electrophoresis
SCUBA	self-contained underwater breathing apparatus
Z-VAD-fmk	*N*-Benzyloxycarbonyl-Val-Ala-Asp(O-Me) fluoromethyl ketone
FACS	fluorescence-activated cell sorting
DMSO	dimethylsulfoxide
