# Supplementary material for: ATG5 Promotes Death Signaling in Response to the Cyclic Depsipeptides Coibamide A and Apratoxin A
Source: Mar Drugs. 2018 Mar 1;16(3):77. doi: 10.3390/md16030077 (PMC5867621; doi:10.3390/md16030077)
Supplement: Supplementary file 1 [file marinedrugs-16-00077-s001.pdf]

Supplementary Material

ATG5 Promotes Death Signaling in Response to the Cyclic Depsipeptides Coibamide A and Apratoxin A

Xuemei Wan [1], Jeffrey D. Serrill [1], Ian R. Humphreys[1], Michelle Tan[1], Kerry L. McPhail[1], Ian G. Ganley[2] and Jane E. Ishmael[1*]

[1] Department of Pharmaceutical Sciences, College of Pharmacy, Oregon State University, Corvallis, Oregon 97331, USA
[2] MRC Protein Phosphorylation and Ubiquitylation Unit, School of Life Sciences, University of Dundee, Dundee, Scotland, UK
* Correspondence: jane.ishmael@oregonstate.edu; Tel.: +01-541-737-5783

Calculation of Cell Doubling Time in Cultured MEFs

MEFs were seeded in a 6-well plate (Corning) with 24,000 cells/well. Cell doubling time was calculated when cells were in the log phase of growth: DT = T ln2/ln(Xe/Xb)
Where:

T = incubation time in any units

Xb = cell number at the start of incubation time

Xe = cell number at the end of incubation time

Results

MEFs ATG +/+

Mean ± SEM 24.1 ± 1.8; N=4

MEFs ATG -/-

Mean ± SEM 19.7 ± 2.4; N=4

Figure S1: **page 2**
Comparison of cellular proliferation and doubling time in untreated wild-type, ATG5-null MEFs.

Figure S2: page 3
Analysis of ATG5 expression in wild-type, ATG5-null and ATG5-null mouse embryonic fibroblasts re-expressing GFP-ATG5.

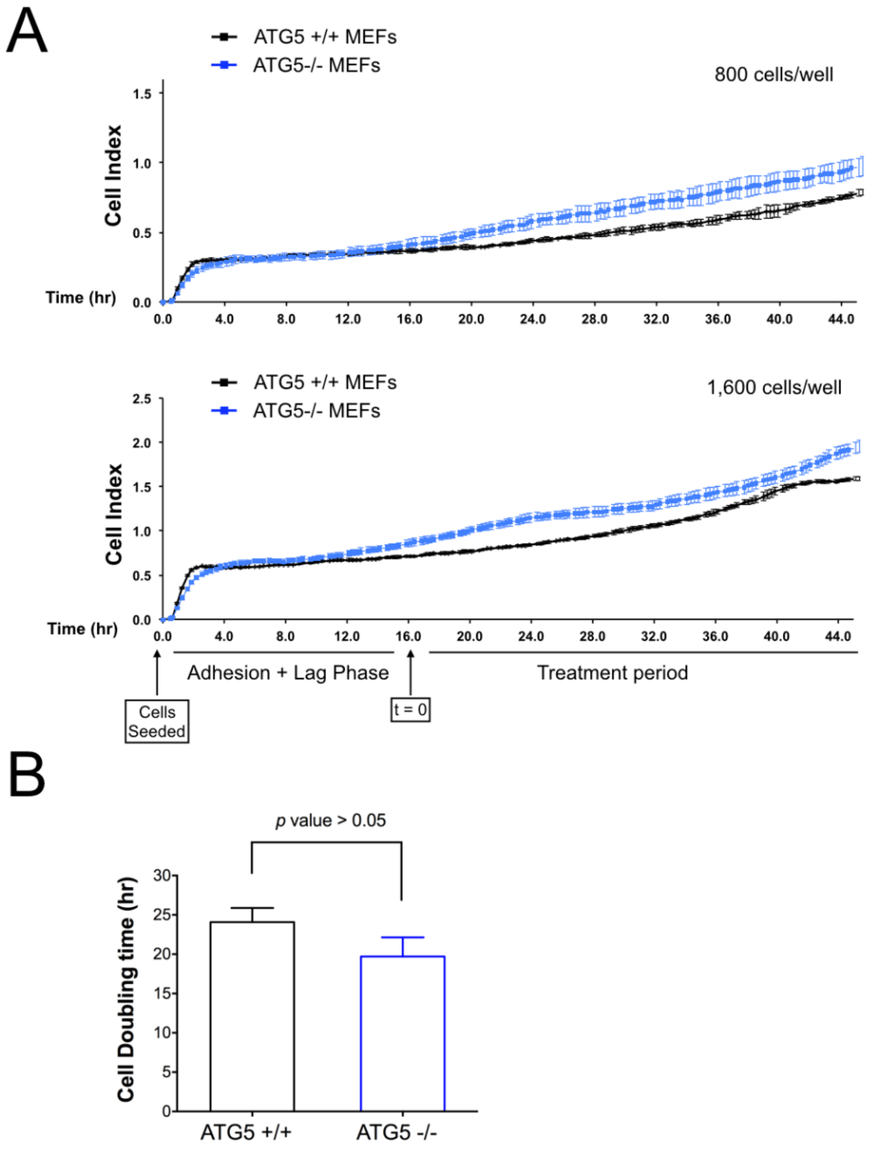

Figure S1. Comparison of cellular proliferation and doubling time in untreated wild-type, ATG5-null MEFs. (**A**) Continuous assessment of cellular proliferation using an xCELLigence real-time cellular analysis (RTCA) DP instrument (ACEA Biosciences, San diego, CA). Wildtype and ATG5-/-MEFs were seeded in E-plate with density of 800 or 1600 cells/well in triplicate in complete medium. Cells were allowed to equilibrate at room temperature for 30 min after seeded. E-plates were inserted into the xCELLigence instrument and impedance measurements were taken at 5 min interval for 8 h, and then at 15 min intervals for the remainder of the study. (**B**) Doubling time (h) of cells used in the study

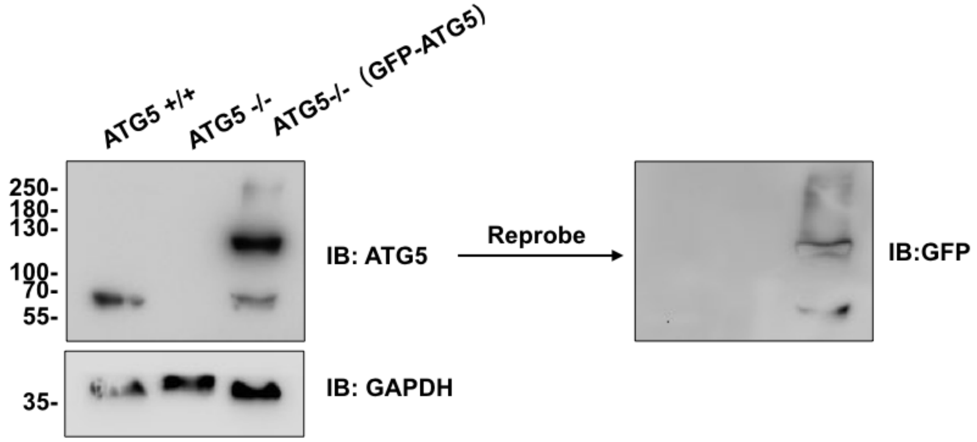

Figure S2. Analysis of ATG5 expression in wild-type, ATG5-null and ATG5-null mouse embryonic fibroblasts re-expressing GFP-ATG5. Immunoblot analysis of ATG5 expression (detected in the context of the covalent ATG5-ATG12 complex) in wild-type and ATG5-/- cells expressing GFP-ATG5. Whole cell lysates were probed with primary antibodies as indicated: anti-ATG5, anti-GFP, and anti-GAPDH.
